# Towards optimal collaboration: reforming the WHO country cooperation strategy in Thailand

**DOI:** 10.2471/BLT.18.219287

**Published:** 2019-07-04

**Authors:** Sirinad Tiantong, Attaya Limwattanayingyong, Suwit Wibulpolprasert, Liviu Vedrasco, Daniel Kertesz

**Affiliations:** a Global Health Division, Ministry of Public Health, Bangkok, Thailand.; b World Health Organization Thailand Country office, Permanent Secretary Building 3, 4th Floor Ministry of Public Health, Tiawanon Road, Nonthaburi 11000, Thailand.

As the leading coordinating agency for global public health, the World Health Organization (WHO) is challenged to improve its country-level work.^1^^,^^2^ In May 2016, the World Health Assembly requested WHO’s senior management to use country budgets and the organization’s social and intellectual capital to leverage additional resources to implement and sustain national programmes.^3^ A 2017 meeting of WHO country representatives highlighted the need to transform WHO into a flexible, nimble, responsive and proactive organization at the country level.^4^ Here we describe how WHO and Thai health authorities (the health ministry, autonomous health agencies outside the ministry, civil society and academia), have created an innovative country cooperation strategy that responds to these recommendations.

At country level, WHO defines its medium-term strategic vision in the country cooperation strategy, a narrative describing how WHO’s offices contribute to the countries’ health priorities. Since 1999, such strategies have been developed for many Member States according to detailed guidelines.^5^ A recent global analysis called for more focused, analytical and strategic country cooperation strategies.^6^

Country cooperation strategies should reflect common elements of strategic planning with a broad outlook and clear, prioritized objectives with acknowledged trade-offs, to identify and catalyse relationships and creativity.^7^ Therefore, a strategic WHO country cooperation strategy should: reflect a process that is managed by and for the Member State; focus on fewer priorities, selected through an evidence-based participatory process; be creative in using WHO’s social and intellectual capital to add value; and innovate to improve collaboration between WHO and the Member State.

Thailand achieved universal health coverage in 2001, and has since worked with civil society, academia and other partners to shape health policies. In addition, the health ministry develops and implements policy alongside several autonomous, partner public health agencies governed by independent boards.^8^

Thailand is the first country to have completed its fifth country cooperation strategy.^9^ Table 1 shows how the strategy has evolved. National health authorities and WHO agreed to focus on fewer priorities and to reconsider the role of WHO in the country to focus on more upstream policy work. During a 9-month consultative process, over 60 stakeholders worked in groups to submit proposals for inclusion as priority areas of the strategy. Proposals were based on specific criteria: relevance to national priorities, potential impact on public health, feasibility and comparative advantage of WHO and other partners participating in implementing the country cooperation strategy. Thirty-eight proposals were considered by the country cooperation strategy Executive Committee, which includes stakeholders from all constituencies. The committee selected six priorities for the 2017–2021 strategy: noncommunicable diseases; road safety; antimicrobial resistance; migrant health; global international trade and health; and global health diplomacy.

**Table 1:** Evolution the WHO country cooperation strategy for Thailand

Country cooperation strategy	Budget/concepts	Advantages	Challenges
2002–2003; 2004–2007; 2008–2011	Budget: WHO only Concept: WHO supports normative functions with many small discrete projects, no clear priority	Financial support distributed to many partners inside and outside the health ministry	Little impact; Substantial management burden
2012–2016	Budget: WHO and Thai health partners both contributing Concept: more focus on big priority programmes; unsuccessful attempt to implement the Paris declaration led to multiple sources of funding, no pooled mechanism and increased transaction costs	More focused approach; Stakeholders’ engagement Initiated by the country	Inadequate engagement with policy-makers; Need more engagement with health ministry
2017–2021	Budget: Mostly domestic funding: WHO 30% and Thai partners 70%; mobilizes more social and intellectual capital from all partners Concept: Big priority programme with evidence-based participatory processes and implementation pooled funding mechanism to reduce transaction costs, consistent with Paris declaration	Sense of ownership and partnership; Expecting greater impact than previous strategies; Streamlined management system; Getting support from the cabinet; Use of social, intellectual and financial capital of both sides	Challenges implementing under uniform financial regulations; Needs more participation, finetuning and discussion at implementation level

WHO: World Health Organization.

These priorities reflect Thailand’s most pressing health issues: more Thais die annually from noncommunicable diseases than from any other cause.^10^ Thailand has enacted legislation designed to reduce road traffic mortality, the ninth highest in the world.^11^ Antimicrobial resistance is both a global and national crisis. Ensuring universal health coverage for migrants and their families is a priority for Thailand, and international trade and health, and global health diplomacy reflect the country’s understanding of the need to intervene globally to improve health locally.

A strategic, focused country cooperation strategy cannot address all health issues. Some areas of work, such as community health, tuberculosis and ageing were not selected as priorities. Some of these issues are addressed through collaboration with other partners or had been sufficiently advanced in previous strategies.

There is no universally accepted definition of country ownership, critical for a successful country cooperation strategy, in the literature. We believe that the Thai Ministry of Public Health owns its country cooperation strategy because the country leads all aspects of the document’s development and implementation. The strategy’s executive committee, convened and chaired by the Permanent Secretary of Public Health, oversees these functions and its secretariat is assumed by the Global Health Division of the health ministry, rather than by WHO.

National health authorities lead implementation of the strategy through six programme subcommittees, one for each priority area, convened and chaired by Thai officials or highly respected senior experts in health. WHO is one of many members in each subcommittee. The health ministry and its country cooperation strategy partners believe that WHO adds value through its intellectual and social capital, rather than through funding. Therefore, the health ministry capitalizes on WHO’s reputation and expertise to engage stakeholders, influence decision-makers and leverage the highest quality technical support available globally.

The Thai country cooperation strategy is catalytic, stimulating both policy dialogue and investment. The programme subcommittees are a fora for multisectoral discussions on the strategy’s priorities. In addition, the Thai country cooperation strategy has mobilized significant domestic funding, with 70% of its  17 million United States dollars five-year budget pledged by the health ministry and the participating partner autonomous public health agencies. We think that the Thai strategy reflects the social, intellectual and financial capital of many agencies and WHO, and for which most resources are domestic. 

WHO, the health ministry and four public health agencies agreed to financially contribute to six un-earmarked pools, one for each priority area. This agreement was inspired by the Paris Declaration on Aid Effectiveness, with the latter’s focus on simplifying procedures and promoting common funding arrangements, and the spirit of trust and collaboration engendered by the country cooperation strategy process. This funding supports a single annual action plan in each priority area, rather than specific activities, which is how, traditionally, WHO funds country-level implementation. WHO and the Thai country cooperation strategy partners have agreed that only flexible, un-earmarked funding will be used for country cooperation strategy programmes, through a letter of agreement approved by WHO Director General, WHO Regional Director for South-East Asia, the Minister of Health, and the boards and directors of each of the participating agencies.

In a formal funding dialogue, partners pledged support against single annual plans and budgets for each priority area. WHO’s contribution was used to address funding gaps in each programme area, but was limited to an agreed 30% of the total country cooperation strategy five-year budget. We expect this mechanism to allow more flexible implementation, reduce transaction costs and encourage participation from other stakeholders, and to encourage other stakeholders to contribute funding, in addition to their intellectual and social capital.

In Thailand, the health ministry and WHO worked for almost two decades to incrementally improve their partnership for greater impact. Concerns about the feasibility of the unique financing mechanism were raised by both the health ministry and WHO when it was first proposed in 2012. Persistent advocacy by the leading negotiators and a thorough discussion of the mechanism’s benefits and risks by all stakeholders ensured that concerns were addressed, and the mechanism was implemented. A system of internal and external monitoring and evaluation, including an independent review of all aspects of the country cooperation strategy planned for October 2019, will help us identify best practices and areas for improvement.

Our approach need not be limited to middle-income countries. Stronger country ownership of country cooperation strategies, establishing fewer priority areas to focus joint work, using WHO’s intellectual and social capital to implement and develop national policy, rather than focusing on funding, are appropriate strategies in any country. Some countries, notably China and Portugal, have established fewer strategic priorities in their country cooperation strategies with WHO, but we believe that Thailand is the first to have catalysed significant domestic resources and used them through a pooled funding mechanism.

The pooled financing mechanism was a significant change in how agencies provide funds and it requires flexibility in adapting administrative procedures. The strategy’s collaborative process also requires time and management flexibility by all stakeholders. Continued involvement of the main stakeholders while streamlining management systems will ensure the strategy’s sustainability.

The Thai WHO country cooperation strategy is an evolving approach to country-level collaboration with WHO. The strategy reflects the deep trust between all partners, and mutual investment of both financial and human resources. WHO, the health ministry and other national health authorities are intent on experimenting and innovating to improve their cooperation strategy, to make progress on Thailand’s most pressing health issues.
